# Barriers and facilitators of linkage to HIV care among HIV-infected young Chinese men who have sex with men: a qualitative study

**DOI:** 10.1186/s12913-017-2158-7

**Published:** 2017-03-16

**Authors:** Haochu Li, Chongyi Wei, Joseph Tucker, Dianmin Kang, Meizhen Liao, Eleanor Holroyd, Jietao Zheng, Qian Qi, Wei Ma

**Affiliations:** 1 School of Public Health, Shandong University, No. 44 Wen Hua Xi Road, Jinan, 250012 China; 2 UNC Project-China, Institute for Global Health and Infectious Diseases, University of North Carolina at Chapel Hill, Chapel Hill, NC USA; 3 Department of Epidemiology and Biostatistics, University of California - San Francisco, San Francisco, CA USA; 4 Shandong Provincial Center for Disease Control and Prevention, Jinan, China; 5 School of Clinical Sciences, Auckland University of Technology, Auckland, New Zealand

**Keywords:** HIV, Linkage to care, Men who have sex with men, China, Qualitative

## Abstract

**Background:**

The Four Free and One Care Policy (HIV/AIDS-related free services) has been in place in China since 2004. However, linkage to human immunodeficiency virus (HIV) care is not yet achieved very well among people living with HIV. We conducted a qualitative study to explore individual and contextual factors that may influence a linkage to HIV care from the perspective of young HIV-infected men who have sex with men (MSM) in a highly centralized HIV care context of China.

**Methods:**

Purposive sampling was used to recruit 21 HIV-infected MSM in Shandong Province, with in-depth interviews conducted between March and July 2015. Thematic content analysis was subsequently used for data analysis.

**Results:**

Key barriers and facilitators related to a linkage to HIV care emerged from participants’ narratives. The barriers included perceived healthy status, low health literacy, and stigma associated with receiving HIV care. The facilitators included an awareness of responsibility, knowledge associated with health literacy, social support, and trusting and relying on services provided by the Center for Disease Control and Prevention (CDC) and the government. These were related to the quality of current HIV counselling and testing, service promotion, and the cost and placement of these HIV services.

**Conclusions:**

In order to improve the MSM linkage to HIV care in China, it is imperative to improve the quality of the current on-going counselling and testing. Further critical linkage support includes increasing supportive services among local CDC systems, designated hospitals and community-based organizations (CBOs), and more financial support for HIV/AIDS related testing, medical checkups and treatments.

## Background

Linkage to care is a critical step in the HIV continuum of care [1]. The World Health Organization (WHO) defines linkage to care as the confirmation of HIV infection or first HIV-specific clinical visit [2]. A growing body of global literature has examined multiple factors related to the linkage to HIV care, which can be classified into three categories, including health care system factors, social factors, and individual characteristics [3–5].

In China, the HIV epidemic has been expanding rapidly among men who have sex with men (MSM), accounting for 17.4% of people living with HIV (PLWH) [6]. In order to reduce HIV infections among Chinese MSM, improved identification of unrecognized infections and timely linkage to care and treatment are critical. A modeling study conducted in China reported that if the testing rate had increased from 50 to 70% and treatment coverage for PLWH had increased to 55% (since 2013), a 25% reduction in annual number of new HIV infections by 2015 might have been achieved [7]. However, many MSM were reported being lost to follow-up at the time of HIV confirmation and cluster of differentiation 4 (CD4) testing. For example, one study found that 21% of MSM who screened HIV-positive did not receive confirmatory testing and 34% of MSM newly diagnosed with HIV/AIDS did not receive CD4 testing within 12 months, posing significant challenges to the test-and-treat strategy [8]. Improving outcomes along the HIV care continuum may also be particularly challenging for certain demographic subgroups such as younger individuals.

A nationwide study of HIV-infected persons in the United States found that significant disparities existed in the continuum of HIV care among different subgroups [9]. There could be a similar situation with young Chinese MSM, due to the high incidence of HIV infection [10] and poor HIV testing uptake being reported [11]. In particular, this subgroup of MSM has not been targeted for HIV prevention in the past. The importance of exploring the issue of linkage to HIV care among these young Chinese MSM is therefore warranted.

In China HIV care is highly centralized with the Centers for Disease Control and Prevention (CDC) in charge of HIV/AIDS related counselling and testing. This is done through cooperation with designated hospitals to provide medical checkups and antiretroviral drugs (ARV) for PLWH [12]. An individual who screens HIV positive and does not have a confirmatory test will not be able to receive free care and treatment services [12]. The CDC system manages the whole HIV care continuum, including HIV screening tests, confirmatory tests, CD4 tests, follow-up after the initial diagnosis, and ARV treatment. In this process, confirmatory testing and CD4 testing after the diagnosis are crucial steps in the linkage to HIV care. The aim of this study was therefore to explore the barriers and facilitators of linkage to HIV care (i.e., uptake of confirmatory testing and CD4 testing after the HIV diagnosis) from the perspective of young adult MSM in China.

## Methods

### Procedure

This qualitative study was conducted in Jinan and Qingdao, two major cities in Shandong Province, China, from March to July 2015. Ethical approval was granted by Institutional Review Board of the University of North Carolina, Chapel Hill; University of California, San Francisco; Shandong University, and Shandong CDC. We defined the linkage to HIV care as completing a confirmatory testing (i.e., Western Blot, WB) or undertaking CD4 testing after the diagnosis. Based on the literature and the findings of our previous studies in China, we refined our semi-structured in-depth interview guide to add questions exploring participants’ local life experience as a tongzhi/gay and being HIV-infected in order to understand their relationships and interaction with peers, friends and healthcare staff. The interview guide featured perception, cognition, and experiences of linkage to HIV care, as well as its related psychosocial, economical, and environmental factors. Main questions included “After you were diagnosed with HIV infection, how did you feel and what did you do?” “In your point of view, what are the advantages and disadvantages of the HIV-related healthcare services, such as confirmatory testing and CD4 testing after the diagnosis?” “Did you receive these HIV-related healthcare services? Why did you receive these services/why not?” “What factors facilitate or impede your access to these HIV-related healthcare services?”

Purposive sampling scheme was adopted, which planned to recruit approximately 20 HIV-infected MSM, including about half of these MSM being linked to HIV care and another half being not linked or facing delays in their linkage to HIV care. The inclusion criteria included: (1) HIV-infected (already existed in the HIV reporting system, or confirmed with a WB, or had a positive result in ELISA), (2) men who reported to having had sex with men in the past 12 months, and (3) aged between 18 and 35 years at the time of HIV diagnosis. According to the above scheme, the local CDC worked with community-based organizations (CBOs) to source eligible participants by way of convenience through their contacts and networks. They contacted 29 potential participants who were eligible, 21 of them agreed to participate, and the other 8 potential participants refused. Potential participants were informed about the current study aiming to understand how they perceived and/or experienced HIV-related healthcare services, in particular, the uptake of confirmatory testing (i.e., WB) and CD4 testing after the diagnosis of HIV infection. After potential participants agreed to participate, appointments were set up and the interviews were arranged in a private room in the local CDC or CBO office. Participants were informed that refusal would not affect their right to use healthcare services, that the interview content would be digitally recorded and transcribed verbatim, and that privacy protection was assured. Written informed consent was then obtained. The first author conducted all of the interviews using mandarin Chinese and participants were informed not to provide their real names, working and home addresses in the interview for the purpose of confidentiality protection. Each interview lasted approximately 90–120 min. In order to compensate for participants’ time spent and transportation costs, we provided each participant 100 RMB (about $16 USD) in cash after completion of the interview. The digital recording was transcribed verbatim in Chinese by student assistants.

### Data analysis

Socio-demographic data were summarized for sample description. We then conducted thematic content analysis on the transcripts. [13] Each interview was briefly summarized immediately after completion, capturing key themes related to facilitators, barriers and suggestions related to linkage to HIV care. Two researchers independently conducted open coding of each transcript using Atlas.ti7 (a qualitative data analysis software). Coding was then compared, and differences were discussed and resolved by group discussions. By using inductive reasoning, factors facilitating or prohibiting linkage to HIV care and suggestions from participants were identified. Relevant and illustrative quotes were grouped together and synthesized. We then developed interpretive memos [14] to better understand why participants received or didn’t receive linkage to HIV care (i.e., finishing WB or undertaking CD4 testing after the diagnosis), which involved individual factors (e.g., HIV/AIDS related knowledge and health consciousness), social factors (e.g., social support), and health care system factors (e.g., limitations in current HIV testing and counselling). The first author translated the quotes into English, and the co-authors crosschecked the accuracy and completeness of the translations.

## Results

### Sociodemographic characteristics

We recruited a total of 21 eligible participants (Table 1), including nine HIV-infected MSM who were linked to HIV care, ten who faced delayed entry into HIV care, and two who were not linked. Among these participants, 52% were aged 18–25, and 48% were aged 26–35. Most of these men (85%) had been first diagnosed as HIV-infected between 2010 and 2014. Most of them (90%) had never married. The majority of these MSM (62%) were registered as local residents. These men were also diverse in terms of education, occupation, and income. Our analysis found the presence of specific barriers, such as worrying about confidentiality, negative attitudes, emotions, psychological burden, and limitations in CDC services (e.g., negative or unfriendly expression and attitudes, lack of guidance and follow-up, clinic time or location inconvenience), and specific facilitators, such as peer referral and accompaniment, and free HIV care, which are in line with the study of Liu and colleagues (2016) [15]. The second level of our analysis then focused on the emergent differential barriers and facilitators in local settings.

**Table 1:** Summary of the characteristics of participants

Characteristics	N (%)
Age, years	
18–25	11 (52.28)
26–35	10 (47.62)
Duration since diagnosis	
~ 2009	2 (9.52)
2010–2014	18 (85.71)
2015~	1 (4.76)
Occupation	
Office worker	6 (28.57)
Service/seller	8 (38.10)
Part time/Self employed	3 (14.29)
Student	3 (14.29)
Unemployed	1 (4.76)
Education	
Postgraduate	1 (4.76)
College	7 (33.33)
High/technological school	7 (33.33)
Secondary school	5 (23.81)
Primary school	1 (4.76)
Annual income (USD)	
More than 9,677	3 (14.29)
4,839–9,677	9 (42.86)
Less than 4,839	4 (19.05)
No income	5 (23.81)
Sexual identity	
Homosexual	16 (76.19)
Bisexual	5 (23.81)
Marriage	
Married	1 (4.76)
Divorced	1 (4.76)
Never married	19 (90.48)
Linkage to HIV Care	
Linked	9 (42.86)
Delayed	10 (47.62)
Not linked	2 (9.52)
On ART	9 (42.86)
Household	
Jinan	4 (19.05)
Qingdao	9 (42.86)
Non local household	8 (38.10)

Note. RMB = Renminbi yuan; $1 U.S. = 6.20 RMB in 2015; ART = antiretroviral therapy

### Barriers to linkage to HIV care

Three major barriers to linkage to HIV care were identified: (1) perceived healthy status; (2) low health literacy; and (3) stigma associated with receiving HIV care.

#### Perceived healthy status

Some participants reported feeling in a good physical condition with no health concerns as a justification for not linking to HIV care. For example, one participant (JN03) said: “When I was diagnosed as HIV-infected, I didn’t want to come out of this place, taking CD4 testing and so on. I felt good physically. Nothing happened. It should be no problem. …I felt very young, very healthy, and did not want to accept [the services in] CDC and did not want to come here.” Another participant (QD08) also described a similar view: “In terms of physical condition, there’s no big problem in my body, just the same as before, very good, no difference. I think it was not necessary [to visit CDC].” In this regard, some of these HIV-infected MSM interviewed, in particular younger informants, usually perceived themselves to be in a good health and therefore did not take their infection of HIV seriously. This misconception may have been related to low health literacy levels.

#### Low health literacy

Many participants lacked a satisfactory knowledge that enabled them to get access to HIV care, such as what services, where to get access, benefits and potential risks. This resulted in ignorance of dealing with their illness, or made them confused and scared. For example, one participant (JN05) described: “I was confused. What’s CD4? What’s viral load? What medicine should I take? Why to take medicine? What benefit? I had no idea at all. … Then, I ran away. Anyway, I felt very scared at that moment.” Some HIV-infected MSM appeared to have a low health literacy about HIV/AIDS and its related services and therefore rejected free HIV care provide by the CDC.

Another participant (JN02) described: “She [a CDC staff] didn’t say anything at all on the phone, just asking me to take my ID card to make a diagnosis. Why should I have a diagnosis? What’s the difference between a diagnosis and non-diagnosis? Because I had no idea of CD4 testing. I thought, when I really get sick, it should not be too late to take medicines. I therefore just stayed at home, and didn’t think about it at all.” In this regard, due to a low health literacy, some HIV-infected MSM may regard HIV/AIDS as a general disease, which should only be treated when feeling sick.

#### Stigma associated with receiving HIV care

The HIV care provided in the CDC were perceived as negative by some participants, because it was a setting associated with HIV/AIDS, a highly publically stigmatized disease. This perception has also been labeled as “courtesy stigma”, a type of stigma by association [16]. For this reason, some participants did not visit the CDC at all. A MSM (JN04) explained: “I didn’t visit CDC, because I had a feeling of disgust towards this kind of place. It’s a feeling of…having this disease is very shameful.” In this regard, the health care setting related to HIV/AIDS services was perceived negatively, resulting in some HIV-infected MSM avoiding these settings.

These MSMs’ negative perceptions of the CDC health care settings could well escalate further anxiety associated with attendance and concordance with care. For example, one participant (JN03) described: “I didn’t know much about this disease. I didn’t want to visit this kind of place [CDC] and test for CD4. It’s jumbled. I was afraid of encountering acquaintances, feeling awkward. Furthermore, this disease is so…[stigmatized].” In this regard, some HIV-infected MSM held negative perceptions of the services provided in the CDC setting and thought they may encounter embarrassing situations.

### Facilitators of linkage to HIV care

Several facilitators to HIV care were identified, including: (1) an awareness of responsibilities; (2) knowledge associated with health literacy; (3) social support; and (4) trusting and relying on CDC services and the government.

#### Awareness of responsibilities

Many participants who were linked to HIV care expressed their sense of self responsibility. For example, one participant (JN01) said: “It’s about how you look at yourself. … You went out to have sex and got infected. It’s your own behavior. You should take your own responsibility, right!” Some HIV-infected MSM also realized their responsibilities to their families and friends. Another participant (QD09) also said: “It’s mainly responsible to my own health, both physically and psychologically, and it’s also responsible to my friends and families.” He elaborated further: “It’s hard to say taking responsibility. It’s about not doing too much harm to them. After all, closer cooperation to services results in a better life. Families and friends would worry less. Poor cooperation would easily result in transmission to others.” In this regard, some HIV-infected MSM realized that linkage to a continuum of HIV care would diminish harm and improve their lives.

#### Knowledge associated with health literacy

Some participants reported when they had knowledge about HIV/AIDS and associated self-care, they actively took part in HIV care (e.g., CD4 testing). For example, a participant (QD10) said: “I realize knowledge is very important. … I insist in taking a CD4 test every three months. …Because I think, if there is a change of the value, I can see it in every three months.” Another participant (JN07) also expressed his view that “It’s for my own health. Anyway, I feel that I have to do the test twice per year, and it’s for my own health. It’s good if nothing happens. But if there’s something going on, I will be informed timely.” These HIV-infected MSM with a sound knowledge about HIV/AIDS and health literacy of self-care were able to monitor their own health through actively engaging in the HIV care.

#### Social support from multiple sources

Some participants reported obtaining social support from friends and work force peers. For example, friends encouraged and reminded them to receive HIV care, thereby providing psychological and spiritual comfort. A participant (JN04) described: “My friend helped me a lot. … He reminded me some things that I didn’t know. … He told me some things that were helpful for me. It’s a kind of comfort, improving me. … At least, I obtained comfort spiritually. Sometimes if only me, I really didn’t want to come [to CDC]”. Friendships provided multi-functional support, not only reminding him but also supporting him both spiritually and psychologically. Another participant (JN05) shared similar experiences describing: “He [a friend] enlightened me. We chatted, had food and played together. … Gradually I calmed down. … He has a lot of knowledge and explained to me. …Gradually I got to know that it’s not so terrible, and then I changed slowly. … I trusted him and thought it should be nothing more than a diagnosis. …At that time I have accepted it and tried to have a change.” Friends and peers were able to not only share HIV related knowledge and information about services, but also provide companionship, which is crucial for HIV-infected MSM to link to HIV care.

Of critical importance for these MSM was the obtaining of personal care and support from healthcare professionals even out of office hours. A participant (JN02) said: “The doctors here get along very well with patients like us. The doctor spent time talking and chatting with us. He taught me to make tea using honey and lemon together, which does a lot of good in our body. He told me a lot of relevant knowledge.” This positive interaction between healthcare professionals and HIV-infected MSM establishes a patient/doctor rapport. Moreover, QQ (a widely used online media platform in China) has been used by local CDC to set up support groups for HIV-infected individuals. Another participant (QD05) who was a member of a QQ support group described: “For HIV-infected individuals like us, the services provided by CDC are currently imperative. They really help us a lot. Without their help, we perhaps are completely filled with questions and do not know where to start. We do not know how to take treatments. With their help, we can at least know when to take medicine, when to do testing, such as CD4 and viral load testing. In this regard, their care is very helpful for us.”

One strength of CBO is being close to the MSM communities and able to provide personalized services to specific HIV-infected MSM. A participant (QD03) recalled: “XX (a CBO member) stated ‘Don’t worry too much, CDC has been doing this work for a long time, they perform well in protecting confidentiality. He then talked to me for a while. I then agreed [to conduct a diagnostic test in CDC]’.” The CBO provided psychological counselling to these men, and accompanied them to go through the linkage process, which made these men feel they were supported and encouraged. Another participant (QD05) described: “Before taking a test, CBO will provide you with psychological counselling. When people feels relaxed and no longer afraid of AIDS, the CBO will then provide you with a HIV test. … If you let me do it by myself, I'm definitely not going to do so. If I go to do it by myself, I feel afraid. To come here (CBO), no matter to do a testing or not, at least someone will accompany me, giving me spiritual support, cheering me up and giving me encouragement.”

#### Trusting and relying on CDC services and the government

Many participants trusted and relied on the CDC and the government. For instance, a participant (JN06) described: “Now there is a CDC staff taking care of us. At least there is a leader in our circle. If we have any questions, we can ask the doctor and he will give us answers. At least we are not alone. … If nobody manages us, we will be very blind and alone. If things turn upside down and disorderly, our illness will become more and more serious. If there are any complications, the doctor can help us to control it in advance. I feel hope anyway.” These men accepted the HIV/AIDS-related services from CDC. Some participants also expressed an expectation of greater financial support for medical checkups and treatment, more service sites, as well as better training and support for CBOs. Another participant (JN01) said: “The government has provided us with free medication and all of the services provided by the government are free. … There is no heavy economic pressure. … This only aims to make us live healthy. There is no reason to refuse cooperation.”

## Discussion

Few qualitative studies have investigated issues related to the linkage to HIV care from the perspective of PLWH in China, in particular delayed diagnosis, late entry care after a HIV diagnosis, and factors influencing the decision to seek care [17, 18]. In China one study to date has examined barriers and facilitators of the linkage to and engagement in HIV care among HIV-infected MSM [15]. However, our study goes further to explore HIV confirmatory testing and CD4 testing after a HIV diagnosis. We contend that this data contributes to an enhanced understanding of linkage to HIV care in the highly centralized context of China. We found that health literacy, stigma associated with receiving HIV care, an awareness of responsibility, social support, and trusting to relying on the CDC and government were important issues in linkage to HIV care. These issues were related to the quality of current HIV counselling and testing, service promotion, and the cost and location of these HIV services.

Our findings contend that the quality of current HIV counselling and testing needs to be improved. Our participants testified to having a lack knowledge of HIV care and their own health status, were worried about stigmatizing settings in the CDC and perceived multiple barriers to accessing HIV care (e.g., misunderstanding, stigma, isolation, psychological problems). As a result, some HIV-infected MSM reported not linking into the formalized HIV care available (e.g., not finishing WB or CD4 testing). This was seen by our informants to be related to the limitations of current HIV counselling and testing, in particular, the pre-test and post-test counselling services. Researchers have argued that HIV diagnosis alone is not enough to ensure that PLWH will get adequate and timely health care, and therefore ask for greater emphasis on the core value of linkage to HIV care in counselling and testing recommendations [19]. Counselling immediately after HIV testing increases rates of successful referral to follow-up care [20]. This counselling needs to be integrated with psychological counselling, in an attempt to address negative attitudes, emotions or mental health issues (e.g., depression, substance abuse) which are significant predictors of poor linkage to HIV care [21].

It is also crucial to provide more training for HIV counselors, which contribute to high rates of linkage to HIV care [22]. In such training, counselors should focus on maximizing HIV-infected MSMs’ capacity to obtain contemporary knowledge on HIV/AIDS and improve health consciousness. Furthermore, in order to maximize HIV counselling and testing, it is important to provide more service hours on a routine basis, and to strengthen privacy of information rights of HIV-infected MSM (e.g., providing one-on-one services in a private room).

Beside the suggested improvements to local HIV counselling and testing, it is also important to provide support services to promote linkage to HIV care. Our analysis indicates that social support from peers, families and friends, healthcare professionals, and CBO services promotes the linkage to care for these young adult HIV-infected MSM. This is consistent with studies arguing that support services, such as case management, outreach, health navigator, mental health services, motivational interviewing, and support groups or educational activities, are crucial in improving linkage to HIV care [23, 24]. Given the characteristics of young adult MSM with HIV, it is necessary to focus on assisting these men’s engagement in the aforementioned support services. Motivational interviewing, for example, could enable HIV-infected MSM to be motivated to take care of themselves, and to see linkage to HIV care as a coping mechanism [25]. Peer networking could be utilized for psychosocial support, healthy living, and preventing transmission of the virus to others [26]. A recent review called for a more coherent, expansive care agenda, which should include the establishment of systems to help households and communities facilitate linkage to HIV care [27]. Young adult MSM generally have close interactions with their peers, and CBOs are better able to link young adult MSM to confirmatory testing and subsequent CD4 testing [28]. In this regard, improving the closer cooperation among CDC systems, communities of MSM and PLWH, and HIV-infected peers is crucial for maximizing linkages to HIV care.

The cost and location of HIV/AIDS-related services are also an important concern. The analysis shows that many HIV-infected MSM accepted the health management of HIV/AIDS in China’s CDC system and showed a willingness in trusting and relying on the government. This was expressed as expecting more service sites in the CDC system and designated hospitals, more financial support in HIV/AIDS related medical checkups, and more training and financial support for effective CBOs. Government support is crucial in linking these men to HIV care, because having public medical insurance and being self-funding were reported to be negatively associated with linkage to HIV care [29]. It has also been reported that HIV clinic-based approaches are cost-effective at linking HIV-infected MSM to care [30]. Initiation of care was most timely when diagnosis occurred at a testing site that also offered medical care, and improving the linkage in these sites will have the greatest effect on timely initiation of care [31]. For those non-medical sites, it is critical to improve referral rates.

The current study is subject to some limitations. Firstly, the themes analyzed were limited by the extent to which individuals disclosed the issues that were relevant and important to them in a comparatively short time. Secondly, the sample was small and the sampling of participants was limited to social networks of local CDC and CBO. Thirdly, it was difficult to recruit HIV-infected MSM who had refused to contact the CDC or the CBO. As an alternative strategy, we recruited HIV-infected MSM who had refused to undertake WB, or lost to follow-up for CD4 testing. Finally, since this was a context specific qualitative study, there are associated transferability concerns.

## Conclusion

The results have advanced knowledge regarding the barriers and facilitators related to linkage to HIV care among HIV-infected young adult MSM in contemporary China. In order to improve the MSM linkage to HIV care in China, it is imperative to improve the quality of the current on-going counselling and testing. Further critical linkage support includes increasing supportive services among local CDC systems, designated hospitals and community-based organizations (CBOs), and more financial support for HIV/AIDS related testing, medical checkups and treatments.

## Abbreviations

AIDS: Acquired immune deficiency syndrome
CBO: Community based organization
CD4: Cluster of differentiation 4
CDC: Center for disease control and prevention
ELISA: Enzyme-linked immuno sorbent assay
HIV: Human immunodeficiency virus
MSM: Men who have sex with men
PLWH: People living with HIV
RMB: Ren min bi
USD: United states dollar
WB: Western blot
WHO: World health organization
